# Host genotype by parasite genotype interactions underlying the resistance of anopheline mosquitoes to *Plasmodium falciparum*

**DOI:** 10.1186/1475-2875-4-3

**Published:** 2005-01-11

**Authors:** Louis Lambrechts, Jean Halbert, Patrick Durand, Louis C Gouagna, Jacob C Koella

**Affiliations:** 1 Laboratoire de Parasitologie Evolutive, CNRS UMR 7103, Université P. & M. Curie, CC 237, 7 quai St Bernard, 75252 Paris cedex 05, France; 2 Génétique et Evolution des Maladies Infectieuses, UMR CNRS-IRD 2724, Centre de Recherche IRD, 911 Avenue Agropolis, BP 64501, 34394 Montpellier Cedex 5, France; 3 Mbita Point Research and Training Centre, International Centre for Insect Physiology and Ecology, PO Box 30, Mbita, Kenya

## Abstract

**Background:**

Most studies on the resistance of mosquitoes to their malaria parasites focus on the response of a mosquito line or colony against a single parasite genotype. In natural situations, however, it may be expected that mosquito-malaria relationships are based, as are many other host-parasite systems, on host genotype by parasite genotype interactions. In such systems, certain hosts are resistant to one subset of the parasite's genotypes, while other hosts are resistant to a different subset.

**Methods:**

To test for genotype by genotype interactions between malaria parasites and their anopheline vectors, different genetic backgrounds (families consisting of the F1 offspring of individual females) of the major African vector *Anopheles gambiae *were challenged with several isolates of the human malaria parasite *Plasmodium falciparum *(obtained from naturally infected children in Kenya).

**Results:**

Averaged across all parasites, the proportion of infected mosquitoes and the number of oocysts found in their midguts were similar in all mosquito families. Both indices of resistance, however, differed considerably among isolates of the parasite. In particular, no mosquito family was most resistant to all parasites, and no parasite isolate was most infectious to all mosquitoes.

**Conclusions:**

These results suggest that the level of mosquito resistance depends on the interaction between its own and the parasite's genotype. This finding thus emphasizes the need to take into account the range of genetic diversity exhibited by mosquito and malaria field populations in ideas and studies concerning the control of malaria.

## Background

In the last few years, exciting advances in the biology and molecular genetics of the development of *Plasmodium *parasites in their mosquito vectors [1,2] have led to the creation of transgenic mosquitoes that are partially resistant to malaria infection [3], bringing the efforts to control malaria with the techniques of transgenesis a major step forward [4,5]. A crucial aspect of these advances is, of course, the fact that the mosquito's genetic make-up determines, at least partly, its resistance to malaria infection [6,7], giving hope for the possibility that key genes controlling resistance may be identified. This hope has been reinforced by the recent identification, in a rodent model of malaria, of several mosquito immune genes that affect parasite development [8,9]. Unfortunately, several aspects of the current knowledge make it difficult to estimate the relevance of such laboratory-based studies to control malaria in natural conditions [10]. One of the crucial aspects is that most studies on the genetics of resistance have considered the response of a mosquito line or colony to a single malaria genotype, while any malaria control programme based on the release of resistant mosquitoes in highly endemic areas can be effective only if mosquitoes are resistant to all the genotypes of the parasite [11]. Because of the limited genetic variation in laboratory colonies compared to natural populations of mosquitoes [12] and the large diversity of natural populations of malaria parasites [13], it is currently far from clear whether this will be possible. One potential problem of the genetic diversity in natural populations could be that, as in many other host-parasite systems (e.g. plant-fungus [14], snail-schistosome [15], bumble-bee-trypanosome [16], *Daphnia*-bacterium [17]), the outcome of the interaction is determined by an interaction between host and parasite genotypes. In systems governed by such genotype by genotype interactions, individual hosts are resistant to only a portion of the parasite genotypes and, reciprocally, individual parasites can infect only particular host genotypes [18]. In other words, no parasite is best at infecting all hosts, and no host is best at resisting all parasites, so that the success of infection depends on the specific combination of the two opponents.

Despite its potentially important role for malaria epidemiology and control, such a genetic specificity of host-parasite compatibility between malaria parasites and their insect vectors have never been investigated. This study examines the potential for genotype by genotype interactions in the combination that is most important for the epidemiology of malaria – *Plasmodium falciparum *and *Anopheles gambiae*. Malaria genotype by mosquito genotype interactions were tested with a standard procedure of quantitative genetics from measurements of the resistance of different genetic backgrounds of the mosquito *A. gambiae*, a major malaria vector in sub-Saharan Africa, to different isolates of the human malaria parasite *P. falciparum*. The parasite isolates were obtained from naturally infected children in western Kenya that harboured gametocytes, the infective stage of the parasite. The genetic backgrounds of mosquitoes were 'mosquito families' generated as the F1 offspring of single egg-laying females. Each mosquito family was challenged with each parasite isolate, and all mosquitoes were simultaneously fed on the blood of the gametocyte carriers via membrane-feeding. This basic design was repeated three times throughout three successive experimental blocks that involved different families and isolates, giving a total of 18 mosquito families, 11 parasite isolates, and 62 specific interactions. As in previous studies [7], the resistance of mosquitoes was quantified with the proportion of blood-fed females that developed oocysts and with the number of oocysts. The genotype by genotype interaction on mosquito resistance was estimated according to standard quantitative genetic methods as the interaction effect in a statistical analysis between the parasite isolate and the mosquito family [17,19]. These methods are based on the idea that sibs are genetically more similar that non-sibs. Therefore, partitioning the variance of any trait (e.g. number of oocysts) among families (individuals sharing a mother) and within groups of sibs give an indication of the extent to which the trait has a genetic basis [20].

## Methods

### Mosquitoes

The mosquitoes used in this study came from a colony that had been established in 2001 from *A. gambiae s.s*. caught in the area surrounding Mbita, a small village on the shore of Lake Victoria in Suba District (western Kenya). These mosquitoes had been initially adapted to feed on a Parafilm^® ^membrane, and then maintained in standard insectary conditions using a rabbit as a blood source for routine maintenance. Females of the colony were blood-fed on a rabbit and allowed to lay eggs in individual vials. Immediately after hatching, each larva was individually placed in one well of a 12-well plate with three mL of filtered lake water. They were fed daily on a standard diet of Tetramin^® ^fish food (0.06 mg per larva on day 0; 0.12 mg on day 1; 0.24 mg on day 2; 0.36 mg on day 3; 0.48 mg on day 4; 0.6 mg on the following days). Adults were kept in an insectary and supplied with a 6% glucose solution and cotton soaked with distilled water. The temperature and humidity in the insectary followed the daily environmental fluctuations. So that mosquito age at pupation did not affect the success of infection, only females that pupated seven days after hatching were used. The wing length of the mosquitoes, measured from the tip (excluding the fringe) to the distal end of the allula with a precision of 0.02 mm, was used as an indication of body size [21]. Where both wings could be measured, the mean of the two lengths was used.

### Gametocyte carriers

*P. falciparum *carriers were recruited from the two- to 10-year old children in the rural area around Mbita, from December 2003 to January 2004. Finger-prick blood samples were collected and thick blood smears were air-dried, stained with 8% Giemsa during 15 minutes, and examined microscopically for the presence of *P. falciparum*. Children with asexual parasitemia (>1,000 parasites/μL) were immediately treated with sulfadoxine-pyrimethamine according to national guidelines. Asymptomatic gametocyte-positive children were recruited for the study after their parents or guardians had signed an informed consent form. The Kenyan and the United States National Institute of Health ethical review committees approved this recruitment procedure.

### Experimental infections

For logistic reasons, the experiment was repeated three times, and within each experimental block infections were done simultaneously. For each of the three blocks, *P. falciparum *isolates were collected from gametocyte carriers that had been identified one or two days before, and used to feed the mosquitoes on the same single day (block 1: December 14, 2003; block 2: January 23, 2004; block 3: January 28, 2004). The gametocyte densities were assessed just before blood withdrawal on a blood smear (as described above) by counting against 500 leukocytes, and converted to numbers of parasites per μL by assuming a standard leukocyte count of 8,000/μL. Although gametocyte densities in the venous blood and the peripheral finger-prick blood might differ, potential differences were assumed to be proportional among the isolates. A sample of five mL of venous blood was collected from each gametocyte carrier in a heparinized tube, 400 μL of which were stored at -20°C for further parasite genotyping. So that the importance of human factors such as transmission-blocking immunity [22] was reduced, the blood was centrifuged at 37°C for three minutes at 2,000 *g *and the autologous serum was replaced with the same volume of a pool of AB serum from two French blood donors without any malaria exposure (the same pool of AB serum was used for all experimental blocks). The mixture was used to feed mosquitoes, which had been starved for 12–16 h before blood feeding, with a standard membrane-feeding system [23]. For each mosquito family, i.e. each group of mosquitoes that was derived from the eggs of a single female, equal groups of three-day old females were randomly chosen and fed separately with each isolate. Depending on the size of the family, each feeding cage contained between four and 15 females. Mosquitoes were allowed to take a blood meal for 40 minutes, after which unfed and partially fed mosquitoes were discarded. Seven or eight days after the infective blood meal, mosquitoes were dissected and their midguts were stained with 2% mercurochrome in distilled water in order to detect the presence and number of oocysts by light microscopy.

### Microsatellite genotyping

*P. falciparum *DNA was extracted from the blood samples using the QIAamp DNA blood kit following the manufacturer's instructions (Qiagen, CA). The isolates were typed using the semi-nested PCR method slightly modified from a previous study [24] (details are available upon request to PD) and the markers used [25] and their GenBank accession number in parenthesis are as follows: PJ2 (G37826), UIDG (G37823), Polyα (L18785), TA60 (AF010556), ARA2 (G37848), Pfg377 (L04161), PfPK2 (X63648), TA87 (AF010571), TA109 (AF010508). The microsatellite PCR products were size-genotyped using a standard size Genescan 500 LIZ on an ABI Prism 310 Genetic Analyser (PE Applied Biosystems, CA).

### Data analysis

Only those mosquito families in which at least four individuals had been fully fed and had survived infection with each isolate were included in the analyses. The likelihood that a mosquito had been infected was analysed with a nominal logistic analysis. The intensity of infection was analysed with an analysis of variance (ANOVA). In this analysis, the square root of the number of oocysts was used, so that the assumptions of the statistical tests (in particular, normality of the residuals) were satisified. As the study was run in three successive experimental blocks, both analyses included the effect of block, and the effects of family, isolate and their interaction. The effect of wing length was also included as a potential confounder [26]. As different families and isolates were used in each experimental block, the factors family, isolate and their interaction were nested within block. Block, family and isolate were considered as random factors.

## Results

The three successive experimental blocks involved three, five and three parasite isolates, and nine, four and five mosquito families, respectively. A third (151) of the 455 mosquitoes of the study were infected by *P. falciparum *oocysts, and the number of oocysts in infected mosquitoes ranged from one to 97 (mean 11.0, median 3). The prevalence and the number of oocysts differed among blocks (block effect, Table 1), and were lower in larger mosquitoes (wing length effect, Table 1).

**Table 1:** Statistical analysis of the effects of mosquito family and parasite isolate on the success of infection. The proportion of infected mosquitoes (a, nominal logistic analysis) and the square root of the number of oocysts (b, ANOVA) were analysed as a function of the mosquito family, the parasite isolate, and their interaction. In both analyses, the mosquito's wing length was included as a confounder. As the study was run in three experimental blocks using different families and isolates, the factors family, isolate and their interaction were nested within block. Block, family and isolate were considered as random factors.

		(a) Proportion infected		(b) Intensity of infection		
Source	d.f.	χ^2^	P	Sum of Squares	F	P

Experimental Block	2	85.5	<0.001	265.2	2.40	0.155
Wing Length	1	3.1	0.029	6.4	7.14	0.008
Family (within Block)	14	3.8	0.927	29.5	0.66	0.800
Isolate (within Block)	8	15.5	0.482	462.5	19.40	<0.001
Family*Isolate (within Block)	34	127.2	<0.001	110.2	3.59	<0.001
Error (for analysis b)	395			356.8		

### Family by isolate interaction

**Figure 1:**
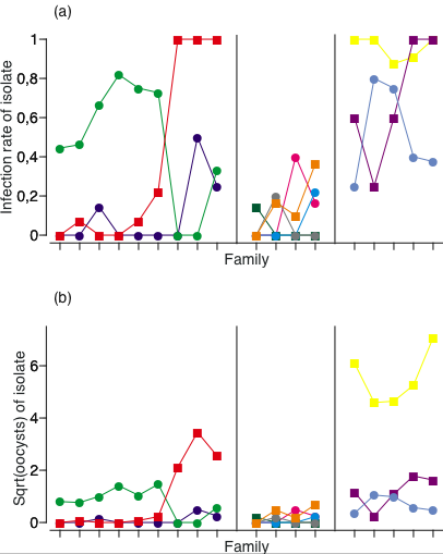
**Graphic representation of the mosquito family by parasite isolate interactions underlying (a) the probability and (b) the intensity of infection**. Each point represents the proportion of infected mosquitoes (in a) or the mean of the square root of the number of oocysts (in b) for a given combination of family and isolate. The families are indicated on the x-axes, and are separated into the three experimental blocks of the study with vertical lines. Different colours represent different isolates (squares: isolates containing two clones; circles: isolates containing three clones), and the lines connect points representing the same isolate. Crossing lines give an indication of family by isolate interactions.

While the crude variation among families (averaged across all parasite isolates) was substantial (mean infection rate ranging from four to 83%; mean number of oocysts ranging from 0.1 to 16.4), most of this variation was due to differences among blocks (Fig. 1), so that there was no evidence that families differed in the overall proportion of infected individuals or in the intensity of infection (family effect, Table 1). Similarly, most of the crude differences among isolates (averaged across mosquito families) were due to differences among blocks. Thus, isolates did not vary in the proportion of mosquitoes they infected (ranging from four to 94%), although they did vary in the number of oocysts they produced (median ranging from zero to 35) (isolate effect, Table 1). More importantly in the context of our study, while neither the families of mosquitoes nor the parasites differed in their average responses to all of their partners, the interaction between mosquito family and parasite isolate (an estimation of the genotype by genotype interaction) had a highly significant effect on the likelihood and the intensity of infection (family by isolate effect, Table 1). Thus, no parasite isolate was most infectious to every host genotype. Rather, isolates that were most infectious on one host tended to be less infectious than the other isolates on other hosts (Fig. 1). Similarly no host genotype was most resistant to every parasite isolate (Fig. 1).

### Genetic characterization of *P. falciparum *isolates

While the quantitative genetic analysis of the data gives an adequate representation of the genetic basis of the mosquito's resistance [20], the use of natural isolates may complicate the interpretation, as (i) they do not necessarily consist of different malaria clones and (ii) isolates often contain several clones in areas where transmission is high [27,28]. However, genotyping the blood samples at nine microsatellite markers showed that the isolates differed. The overall genetic diversity was high, ranging from four to 15 allelic variants per locus. Each isolate had an allelic pattern that differed from all other isolates at, at least, one locus (data not shown), showing that the isolates were genetically distinct. Using the maximum number of alleles at a single locus as a conservative estimate of the number of clones, each isolate was found to contain either two or three distinct clones of *P. falciparum *(Table 2).

**Table 2:** Description of *P. falciparum *isolates. The number of gametocytes per 500 leukocytes, converted to numbers of parasites per μL (assuming a standard leukocyte count of 8,000/μL) and the maximum number of alleles at a single locus found for 9 microsatellite markers (a conservative estimate of the number of clones) are given for each isolate.

Experimental block	Isolate	Gametocyte density (parasites/μL)	Number of clones
1	A	176	3
1	B	32	3
1	C	32	2
2	D	32	3
2	E	16	2
2	F	16	3
2	G	32	3
2	H	32	2
3	I	48	2
3	J	16	2
3	K	16	3

### Potential confounding effects

Separate analyses of the data for the two numbers of clones (two or three) ensured that the number of clones contained in each isolate did not confound the interpretation. The effect of the mosquito family by parasite isolate interaction on the likelihood of infection was significant in both cases (two clones: *P *= 0.050; three clones: *P *= 0.003) and the effect of the interaction on the number of oocysts was significant in one of the cases and showed a tendency in the other case (two clones: *P *= 0.219; three clones: *P *= 0.008).

In addition, differences in gametocyte density between isolates (Table 2) may be expected to bias infection success [23]. There were sufficient data to analyse the effects of the mosquito family by parasite isolate interaction separately for the isolates with 16 or 32 gametocytes/μL (i.e. one or two gametocytes per 500 leukocytes). At both gametocyte densities, the interaction significantly influenced the probability of infection (16 gametocytes/μL: *P *< 0.001; 32 gametocytes/μL: *P *< 0.001) and the number of oocysts (16 gametocytes/μL: *P *< 0.001; 32 gametocytes/μL: *P *< 0.001). In conclusion, the two potential confounders – number of clones per isolate and gametocyte density – had no qualitative influence on the results of the analysis.

## Discussion

While the specificity of mosquito-malaria interactions at the species level is well documented [29], the present results are the first experimental evidence of the genetic specificity of mosquito infection by malaria parasites at the intraspecific level. This finding corroborates an earlier study, where a mosquito line selected for resistance to malaria infection varied considerably in its response against different *Plasmodium *species and strains [6]. The present study goes one step further by suggesting that mosquito resistance to malaria is at least partly determined by the specific interaction between its own and the parasite's genotype. This idea is supported by two other studies showing that, in a strain of mosquitoes selected to resist infection by a wide variety of malaria species, different genetic loci are involved in the responses against different *Plasmodium *parasites [30,31]. The present study suggests, moreover, that the genes conferring resistance to a particular parasite depend on the genetic background of the mosquito.

The specificity of host-parasite interactions is often postulated to occur at the level of parasite recognition. While the molecular mechanisms of *Plasmodium *recognition by mosquitoes are still largely unknown, a group of thioester-containing proteins (TEPs) represents a promising family of candidate recognition molecules. One of them, the complement-like protein TEP1, has recently been shown to bind to and mediate the killing of the rodent malaria parasite *P. berghei *by the mosquito *A. gambiae *[9]. Moreover, two allelic variants of the *TEP1 *gene are associated to susceptible and refractory strains of *A. gambiae *[9]. It is therefore tempting to speculate that this protein may be involved in the specific recognition of particular malaria genotypes by the insect's immune system.

The mosquito genotype by parasite genotype interactions shown in this paper may help to understand some puzzling aspects of the epidemiology of malaria. Thus, even in areas with intense transmission, the probability that a mosquito becomes infected is generally low [26,32]. Furthermore, the probability of infection is generally low even when, as in our study, mosquitoes fed on a blood-meal known to contain infectious gametocytes [33,34]. This could have several explanations: mosquitoes fail to pick up infective gametocytes, transmission-blocking immunity in the human hosts prevents the parasite's development within the mosquito [22,35], or parasites are cleared by mosquitoes that mount a sufficiently effective immune response [2]. The present results indicate an additional reason: that many parasites are incompatible with many of the mosquitoes in a natural population.

The epidemiological consequences of mosquito genotype by malaria genotype interactions are perhaps most obvious in the context of malaria control with mosquitoes transformed to be resistant against malaria. The strong genetic specificity of compatibility between parasite isolates and individual insect vectors suggests that most studies on the mechanisms underlying the resistance of mosquitoes against *Plasmodium *might be misleading for the development of malaria control strategies. Indeed, most of these laboratory-based studies focus on the response of one mosquito line or colony against a single parasite strain and thus do not represent the genetic diversity of mosquitoes and parasites in natural populations [12,13]. However, any malaria control programme based on the release of mosquitoes harbouring 'resistance genes' is unlikely to be effective if resistance is expressed against only a subset of the parasite genotypes of the local population. Indeed, as parasites facing resistant mosquitoes will be under strong selective pressure to avoid mosquito defence mechanisms, genotypes that are eliminated by the resistance genes might be replaced rapidly by genotypes that cannot be controlled. Thus, before a possible release of transgenic mosquitoes, it will be crucial to ensure that the transformed mosquitoes are resistant to all of the parasite genotypes in the local population. This reinforces the idea that any release of genetically modified mosquitoes for reducing transmission of mosquito-borne diseases must be preceded by studies that have moved from the laboratory to the field [10].

## Conclusions

This study demonstrated that the resistance of an anopheline mosquito to *P. falciparum *development, a major component of its vector competence, varies considerably between different combinations of parasite isolates and individual, genetically variable, vectors. Optimal transmission may thus require some specific compatibility between the insect's and the parasite's genotypes. This result has important consequences for the epidemiology of malaria. Overall, it suggests that conclusions from a particular subset of mosquito and malaria genotypes will not necessarily hold for other combinations of genotypes. Therefore, field studies taking into account the full diversity of mosquito and parasite populations are necessary to reach valid conclusions concerning the technologies developed in laboratories for the control of malaria.

## Authors' contributions

LL participated in the design and the coordination of the study, carried out the fieldwork, participated in the molecular analysis, performed the statistical analysis, and wrote the manuscript. JH participated in the fieldwork. PD carried out the molecular analyses and helped to draft the manuscript. LCG supervised and coordinated the fieldwork. JCK conceived and designed the study, performed the statistical analysis, and wrote the manuscript.
